# Surveillance of SARS-CoV-2 antibodies of patients in the local affected area during Wuhan lockdown

**DOI:** 10.1186/s12879-021-07010-w

**Published:** 2022-01-04

**Authors:** Yueting Tang, Jiayu Sun, Yumeng Yuan, Fen Yao, Bokun Zheng, Gui Yang, Wen Xie, Guangming Ye, Zhen Li, Xiaoyang Jiao, Yirong Li

**Affiliations:** 1 Department of Clinical Laboratory, Zhongnan Hospital of Wuhan University, Wuhan, 430071 Hubei China; 2 Center for Clinical Gene Diagnosis, Zhongnan Hospital of Wuhan University, Wuhan, Hubei China; 3 Shantou University Medical College, Shantou, Guangdong China

**Keywords:** COVID-19, SARS-CoV-2, Serosurveillance

## Abstract

**Background:**

Serosurveillance is crucial in estimating the range of SARS-CoV-2 infections, predicting the possibility of another wave, and deciding on a vaccination strategy. To understand the herd immunity after the COVID-19 pandemic, the seroprevalence was measured in 3062 individuals with or without COVID-19 from the clinic.

**Methods:**

The levels of SARS-CoV-2 antibody IgM and IgG were measured by the immuno-colloidal gold method. A fusion fragment of nucleocapsid and spike protein was detected by a qualitative test kit with sensitivity (89%) and specificity (98%).

**Results:**

The seroprevalence rate for IgM and IgG in all outpatients was 2.81% and 7.51%, respectively. The sex-related prevalence rate of IgG was significantly higher (P < 0.05) in women than men. The highest positive rate of IgM was observed in individuals < 20 years of age (3.57%), while the highest seroprevalence for IgG was observed in persons > 60 years of age (8.61%). Positive rates of IgM and IgG in the convalescent patients were 31.82% and 77.27%, respectively, which was significantly higher than individuals with suspected syndromes or individuals without any clinical signs (P < 0.01). Seroprevalence for IgG in medical staff was markedly higher than those in residents. No significant difference of seroprevalence was found among patients with different comorbidities (P > 0.05).

**Conclusions:**

The low positive rate of the SARS-CoV-2 IgM and nucleic acid (NA) test indicated that the SARS-CoV-2 outbreak is subsiding after 3 months, and the possibility of reintroduction of the virus from an unidentified natural reservoir is low. Seroprevalence provides information for humoral immunity and vaccine in the future.

## Abstract (article highlights)

1. Two months after the COVID-19 outbreak in Wuhan, the seroprevalence for IgM and IgG in the convalescent patients was 31.82% and 77.27%, respectively. In the total outpatient population, the mean seroprevalence for IgM and IgG in all participants was 2.81% and 7.51%, respectively.

2. Seroprevalence for IgG in medical staff was markedly higher than those in residents.

3. The seroprevalence for IgG was significantly higher in women and elderly individuals than those in men and younger individuals. Individuals with comorbidity had higher seroprevalence than those without comorbidity.

## Background

COVID-19, caused by severe acute respiratory syndrome coronavirus-2 (SARS-CoV-2), has been the cause of the global pandemic. The latest data reported more than 260 million laboratory-confirmed COVID-19 cases with over 5 million deaths worldwide [1]. The high morbidity and mortality have made it a significant threat to global health [2]. Evidence suggests that SARS-CoV-2 can be transmitted effectively among humans, primarily by asymptomatic carriers through droplets or direct contact [3–6]. The trend of increase rate mostly follows exponential growth. The mean primary reproduction number (R0) was estimated to range from 2.24 to 3.58, be associated with two- to eight-fold increases in the reporting rate, and have an epidemic doubling time of 6.4 days (95% CrI 5.8–7.1 days) [7, 8].

Due to the lack of effective therapy, interrupting the transmission route by finding and isolating patients is an effective measure to control the disease. In the early stage of COVID-19, patients might manifest only transient febrile illness and minimal respiratory illness or be completely free of any clinical symptoms or signs [9, 10]. The sub-clinical individuals may serve as reservoir and become potential source of infection, making it much more difficult to control the disease. Surveillance for infection is usually applied to address the transmission patterns, to observe latent infections, and to analyze disease progression. After strenuous efforts on epidemic control, the newly diagnosed cases have significantly decreased. However, in some areas, a second outbreak has been more severe. Serosurveillance is crucial in estimating the range of SARS-CoV-2 infections, predicting the possibility of another wave, and deciding on a vaccination strategy.

Neutralizing antibodies (NAbs) are critical components in the protective immune responses to viral infections because they can bind to viral particles and block them from entering the host cells [11, 12]. NAbs are essential for protecting populations from re-infection. Information on NAbs could be used to understand the epidemiology of SARS-CoV-2 infection and help determine the level of humoral immunity in patients. To COVID-19, different populations in different regions may have different humoral immunity. Wuhan was the epicenter of COVID-19 in China, with the highest infectious rates; residents who lived in this city should had a high-risk for virus exposure. However, few reports have investigated the residents' infection with certainty. As a tertiary university medical center in metropolitan Wuhan, Zhongnan Hospital has a 3300-bed capacity, and it serves about 100,000 people. Zhongnan Hospital was designated as a hospital responsible for COVID-19 patients’ treatment from the 23rd Jan to 15th Mar 2020 and as a detection institution throughout the pandemic. Many severely and critically ill patients were transferred there for intense therapy. Then, we selected this hospital to study the prevalence of COVID-19 infection. Given the relative extraordinary exposure history of the individuals, including patients and hospital staff, their seroprevalence may provide valuable information about the population infection and their immune status. Seroprevalence of residents is vital for understanding the infectious population scale and their immune status, and preventing disease spread and reemergence.

## Methods

### Sample collection

This study received ethics approval from the Ethics Committee of Zhongnan Hospital, Wuhan University (No. 2020051K) and followed the decalration of Helsinki. Blood samples were collected from a total of 3062 outpatients, including 2597 ordinary patients (outpatients without COVID-19 confirmed history or symptoms related to COVID-19) for COVID-19 screening, 355 individuals with suspected clinical symptoms, and 110 confirmed COVID-19 patients whose diagnosis was defined based on the New Coronavirus Pneumonia Prevention and Control Program (7th edition) published by the National Health Commission of China. Blood samples were obtained in in vacutainer tubes without anticoagulant and kept at room temperature for 30 min to ensure serum separation. Serum samples were collected after being centrifuged at 4000 rpm for 5 min. Whether the participants had been exposed or not, as well as the time of their exposure cannot be known with certainty. Therefore, a single blood sample was taken for antibody testing. Blood samples were collected after March 21, 2020, approximately 2 months after the outbreaks were reported.

### Antibody and nucleic acid testing for SARS-CoV-2 detection

Serum IgM and IgG of SARS-CoV-2 was measured by immuno-colloidal gold technology (INNOVITA Biotechnology Company, Tangshan, China). A fusion fragment of nucleocapsid and spike proteins was detected by the qualitative kit. The sensitivity and specificity of the tests was 89% (95%CI 80.40–92.00%) and 98% (95%CI 94.20–100%), respectively [13]. 10 μL of serum samples was diluted with two drops of sample diluent and then added into sampling well and results were read within 15 min. A positive outcome was indicated when both the control line and the test line appeared simultaneously. Test kits showing only control lines are indicative of negative result, and if the control line does not appear, the result is invalid. At the same time, the nucleic acid (NA) of SARS-CoV-2 was measured by reverse transcription-polymerase chain reaction (RT-PCR) (DAAN GENE Company, Guangzhou, China). Also, all participants underwent a CT scan to confirm whether there were pathogenic lesions in the lung.

### Statistical analyses

For seropositive populations, the positive rates in each group were calculated by the number of positive individuals divided by the corresponding entire tested population. Data were expressed as numbers and proportions. The 95% confidence interval (CI) was presented. Differences in frequencies or proportions were tested using a χ^2^ test. SPSS 20.0 was used for statistical analyses (SPSS Incorporated, Chicago, IL, USA). A P < 0.05 was considered statistically significant.

## Results

### Seroprevalence for IgM and IgG among different groups

In total, 3062 individuals were enrolled in this study, of whom 1652 were males and 1410 were females. Of 3062 samples, the seroprevalence for IgM and IgG were 86 (2.81%) and 230 (7.51%), respectively. Among 268 seropositive individuals, 97 (36.19%) persons has positive CT scan result; only four persons (1.49%) had a positive NA test, including two convalescent COVID-19 patients who recovered in the observation period and two asymptomatic infection cases. The rate of specific antibody IgG was significantly higher in women than in men (P < 0.05).

Seroprevalence for IgM was significantly different among age group. From high to low: < 20 years (3.57%), 41–60 years (3.49%), > 60 years (3.36%), and 21–40 years of age (1.61%) (P < 0.05) (Table 1). Seroprevalence for IgG among age group from high to low was > 60 years (8.61%), 41–60 years (8.24%), 21–40 years (6.14%) and ≤ 20 years of age (3.57%) (P = 0.098) (Table 1).

**Table 1:** Positive rate of SARS-CoV2 among different groups

Characteristics	No. P	No. (%) 95%CI			
		IgM (+)	IgG (+)	NA(+)	CT(+)
*Sex*					
Male	1652	41 (2.48) 1.81–3.38	104 (6.30) 5.20–7.60	1 (0.06) 0.003–0.39	34 (2.06) 1.45–2.90
Female	1410	45 (3.19) 2.36–4.28	126 (8.94) 7.52–10.58	3 (0.21) 0.055–0.68	63 (4.47) 3.48–5.72
P		0.141	0.004	0.256	0.000
*Age*					
≤ 20	56	2 (3.57) 0.62–13.38	2 (3.57) 0.62–13.38	0	0
21–40	1059	17 (1.61) 0.97–2.61	65 (6.14) 4.80–7.80	0	15 (1.42) 0.82–2.38
41–60	1262	44 (3.49) 2.57–4.69	104 (8.24) 6.81–9.93	3 (0.24) 0.06–0.75	49 (3.88) 2.92–5.14
> 60	685	23 (3.36) 2.19–5.07	59 (8.61) 6.67–11.03	1 (0.15) 0.1–0.94	33 (4.82) 3.39–6.77
P		0.035	0.098	0.461	0.000
*Consulting room*					
Fever clinic for patients	493	51 (10.34) 7.87–13.46	117 (23.73) 20.1–27.79	1 (0.20) 0.01–1.31	64 (12.98) 10.21–16.35
Fever clinic for medical staff	16	0	4 (25.00) 8.33–52.59	1 (6.25) 0.33–32.29	3 (18.75) 4.97–46.31
Internal medicine	1395	21 (1.51) 0.96–2.33	70 (5.02) 3.96–6.33	1 (0.07) 0.004–0.46	23 (1.65) 1.07–2.50
Surgery department	659	9 (1.37) 0.067–2.67	18 (2.73) 1.68–4.37	1 (0.15) 0.008–0.98	2 (0.30) 0.05–1.22
Obstetrics/gynecology/fertility	135	1 (0.74) 0.04–4.67	4 (2.96) 0.95–7.88	0	2 (1.48) 0.26–5.79
Pediatric Department	4	0	1 (25) 1.32–78.06	0	0
Oncology department	261	3 (1.15) 0.30–3.60	8 (3.07) 1.43–6.18	0	2 (0.77) 0.13–3.04
General department/other	99	1 (1.01) 0.05–6.30	8 (8.08) 3.81–15.76	0	1 (1.01) 0.05–6.30
P		0.000	0.000	0.000	0.000
*Patient classification*					
COVID-19 convalescent	110	35 (31.82) 23.45–41.48	85 (77.27) 68.11–84.49	2 (1.82) 0.32–7.10	51 (46.36) 36.89–56.09
SSC	355	12 (3.38) 1.84–5.99	28 (7.89) 5.40–11.32	0	14 (3.94) 2.26–6.68
SAC	1295	22 (1.70) 1.09–2.61	74 (5.71) 4.54–7.16	1 (0.08) 0.004–0.5	19 (1.47) 0.91–2.33
Attendance for other diseases	1302	17 (1.30) 0.79–2.13	43 (3.30) 2.43–4.46	1 (0.08) 0.004–0.50	13 (1.00) 0.56–1.75
P		0.000	0.000	0.000	0.000
*Comorbidity*					
Tumor	387	7 (1.81) 0.79–3.86	14 (3.62) 2.07–6.14	0	5 (1.29) 0.48–3.17
CCD	146	2 (1.37) 0.24–5.37	5 (3.42) 1.27–8.22	0	2 (1.37) 0.24–5.37
Digestive diseases	259	2 (0.77) 0.13–3.06	11 (4.25) 2.25–7.68	0	2 (0.77) 0.13–3.06
Urogenital diseases	141	0	3 (2.13) 0.55–6.57	0	1 (0.71) 0.04–4.48
Nervous diseases	51	2 (3.92) 0.68–14.59	2 (3.92) 0.68–14.59	1 (1.96) 0.68–14.59	1 (1.96) 0.10–11.79
Hematological diseases	24	1 (4.17) 0.22–23.12	1 (4.17) 0.22–23.12	0	0
Other respiratory diseases	64	1 (1.56) 0.08–9.54	0	0	1 (1.56) 0.08–9.54
External injury	90	1 (1.11) 0.058–6.90	2 (2.22) 0.39–8.56	0	0
Pregnancy check-up	68	1 (1.47) 0.08–9.01	2 (2.94) 0.51–11.16	0	1 (1.47) 0.08–9.01
AIDS	13	0	0	0	0
Other	59	0	3 (5.08) 1.32–15.06	0	0
P		0.584	0.881	0.006	0.967

*CI* confidence interval; *SSC* screening for symptomatic conditions (the most common symptoms related to COVID-19 including fever, cough, chest tightness, diarrhea); *SAC* screening for asymptomatic conditions (health examination professionals were asymptomatic currently, but did not rule out close contacts or had a symptom related to COVID-19); *CCD* cardiovascular and cerebrovascular diseases; *Other* including autoimmune diseases, skin diseases, stomatitis, laryngeal eyewinker, poisoning, etc.

We then grouped all subjects by their consulting department (Table 1), including Fever Clinic for patients (an outpatient clinic especially for the treatment of febrile or suspected patients), Fever Clinic for medical staff (clinic especially for screening highly suspected medical staff or the treatment of confirmed cases), Internal Medicine, Surgery department, Obstetrics/Gynecology/Fertility Clinic, Pediatric Department, Oncology Department, and other departments. The highest seroprevalence for IgM was observed in the Fever Clinic. From high to low: Fever Clinic for patients (10.34%), Internal Medicine (1.51%), Surgery Department (1.37%), Oncology Department (1.15%), General Department or other departments (1.01%), Obstetrics/Gynecology/Fertility Department (0.74%) (P < 0.001). IgG positive rate (from high to low): Fever Clinic for medical staff (25.0%), Fever Clinic for patients (23.73%), General Department or other departments (8.08%), Internal Medicine (5.02%), Oncology Department (3.07%), Obstetrics/Gynecology/Fertility Department (2.96%), and Surgery Department (2.73%) (P < 0.001) (Table 1). Participants from the Fever Clinic for medical staff and Pediatric Department only had antibodies to IgG during the study.

The criteria for discharging patients was being afebrile for 14 days, clinical improvement, and negative in the NA test. In this study, we also included 110 COVID-19 patients discharged from the hospital to evaluate their immunity. The seroprevalence for IgM and IgG in the patients was 35 (31.82%) and 85 (77.27%), respectively, which was significantly higher as compared with those in other groups (Table 1). Among the 110 confirmed COVID-19 patients, most of the patients were convalescent, and their positive rate of IgG was markedly higher than that of IgM. Fourteen patients were IgM- and IgG-negative, while 11 cases were IgM-positive, but IgG-negative, and maybe were newly infected patients in the early stages (4–6 days post symptom onset) [14, 15]. CT scans showed that 46.36% of patients still had inflammatory lesions in pulmonary interstitial and/or parenchyma, indicating that it takes some time to absorb pulmonary inflammation. Forty-one patients with positive antibodies were conducted with dynamic observation. Among them, 16 IgM-positive patients changed to IgM-negative on an average of 5 days, while six IgG-negative patients changed to IgG-positive in an average of 4.3 days. Three hundred fifty-five individuals with suspected syndromes, including fever, cough, chest congestion, and headache of undetermined origin, were included in the study. The positive rate of IgM and IgG were 12 (3.38%) and 28 (7.89%), respectively. Their NA tests were negative, but 14 cases (3.94%) had lung abnormalities by CT. However, 1295 participants were currently asymptomatic. Still, some of them had recalled they might have had a suspected symptom, without particular diagnosis due to mild symptoms, and chose to stay-at-home in quarantine. Their seroprevalence for IgM and IgG were 22 (1.71%) and 74 (5.74%), respectively. Only one was positive in the NA tests, and 19 (1.47%) cases had lung abnormalities by CT (Table 1).

When we re-classified the participants according to their comorbidities, the 13 AIDS patients tested showed no positive result for the IgM, IgG, NA and CT scan (Table 1). Among 387 tumor patients, 7 (1.81%) were IgM positive, while 14 (3.62%) were IgG positive. One hundred forty-six patients with cardiovascular and cerebrovascular diseases, hypertension, and diabetes (CCD) had an IgM- and IgG-positive rate of 1.37% (2) and 3.42% (5), respectively. Two hundred fifty-nine patients with digestive system diseases had high levels of IgG (11, 4.25%) but low levels of IgM (0.77%). One hundred forty-one patients with urogenital system diseases had levels of IgG (3, 2.13%), but none had IgM. Fifty-one patients with nerve system disease, including coma, syncope, disturbance of consciousness, dizziness, headache, epilepsy, spasm, neuralgia, and Alzheimer’s disease, etc., had IgM- and IgG-positive rates of 3.92% (2) and 3.92% (2), respectively. Twenty-four patients with hematological disease had IgM- and IgG-positive rates of 4.17% (1), and 4.17% (1), respectively. Sixty-four patients with other respiratory disorders, including COPD, asthma, rhinitis, pharyngitis, bronchitis, and other pathogenic lung infections had low IgM- and IgG-positive rates of only 1.56% (1), and 0.00%, respectively. Ninety patients with external injury had IgM- and IgG-positive rates, of 1.11% (1), and 2.22% (2), respectively. Sixty-eight patients for pregnancy check-up had IgM- and IgG-positive rates of 1.47% (1) and 2.94% (2), respectively. At last, other disease including autoimmune diseases, skin diseases, stomatitis, laryngeal eyewinker, and poisoning, etc., had IgM- and IgG-positive rate of 0.00% and 5.08% (3), respectively (Table 1).

### The variation of IgM and IgG in COVID-19 patients

**Fig. 1:**
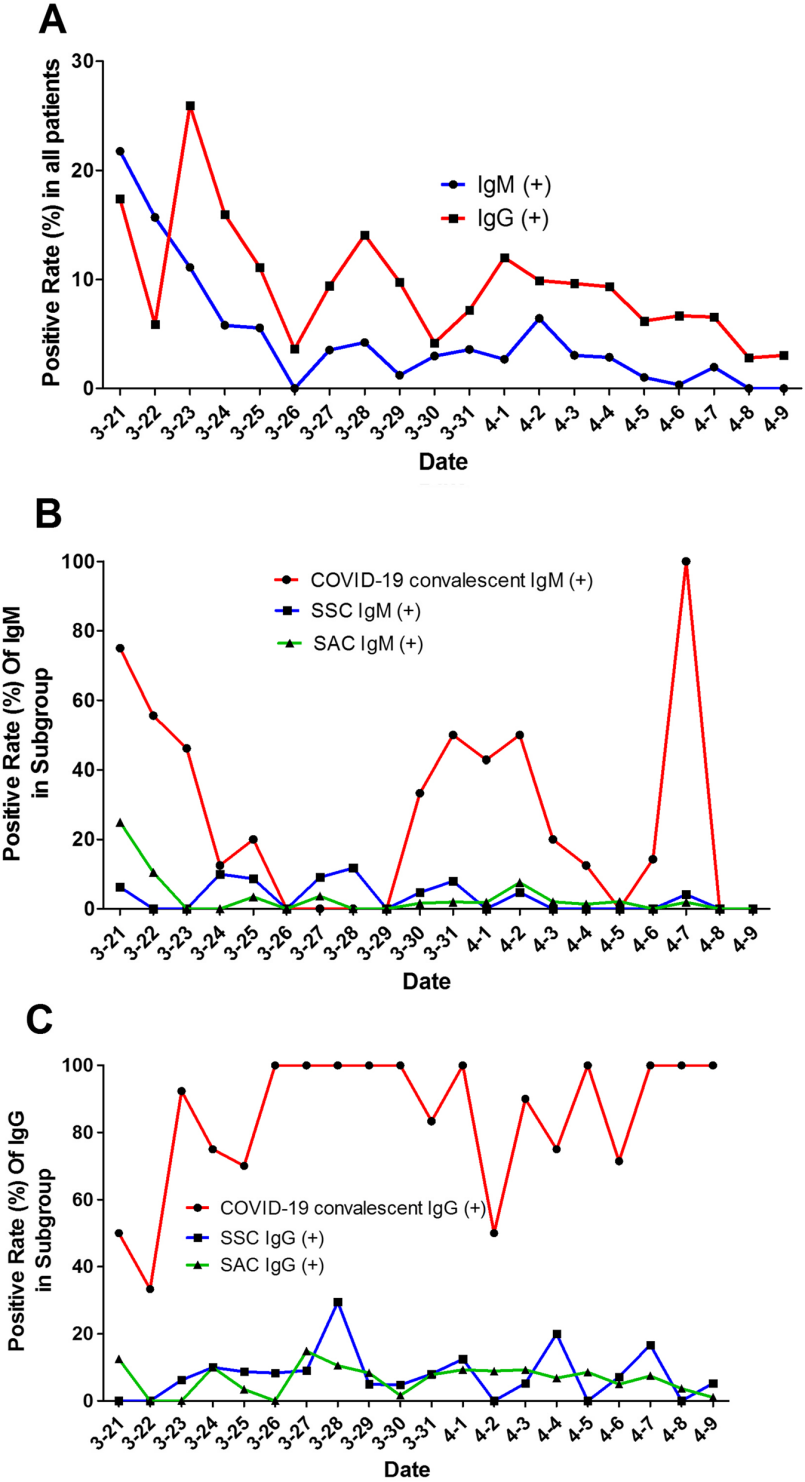
Temporal change of IgM and IgG in COVID-19 patients. **A** The curve shows that positive rates of IgM and IgG in patients were reduced after March 22. The small peak occurring on March 28 is due to the attendance of more COVID-19 convalescent and SAC patients at that time. **B** In general, the positive rate of IgM was higher positive rate in COVID-19 convalescents than the SAC and SCC individuals. **C** During all study periods, the COVID-19 convalescents showed a higher positive IgG rate than the SAC and SCC individuals

The percentage of patients who were IgM-positive became reduced after March 22, and was maintained at a low level after that. Seroprevalence for IgG peaked at 25.93% on March 23 due to many COVID-19 convalescent patients testing positive for NAbs on those days. As time passed, the positive rates of IgG remained at a low level; the range was from 2.82 to 14.08% (Fig. 1A). Most patients were convalescent, and the positive rate of IgG was greater than that of IgM (Fig. 1A). As for subgroups, the positive rate of IgM was at a higher level in the COVID-19 convalescent group (mean ± SE: 33.41 ± 0.34) compared to the SSC [Screening for symptomatic condition (the most common symptoms related to COVID-19 including fever, cough, chest tightness, diarrhea)] group (mean ± SE: 4.41 ± 0.09) and the SAC [screening for asymptomatic conditions (health examination professionals who were asymptomatic currently, but did not rule out close contacts or had a symptom related to COVID-19)] group (mean ± SE: 1.28 ± 0.09) (Fig. 1B). During all study periods, the COVID-19 convalescent group showed a higher IgG-positive rate (mean ± SE: 81.19 ± 0.26) than the SSC (mean ± SE: 7.35 ± 0.05) and SAC group (mean ± SE: 4.62 ± 0.09) (Fig. 1C).

## Discussion

Due to its extremely high contagious nature, COVID-19 poses a significant threat to global public health. It is of utmost importance to know whether the SARS-CoV-2 outbreak is subsiding after tremendous efforts on interrupting the human-to-human transmission to reduce secondary infections among close contacts, and to prevent transmission amplification events. Currently, the first epidemic of COVID-19 is under control in Wuhan. Much subsequent work should be on preventing asymptomatic transmission. Some asymptomatic individuals might still exist. serving as a reservoir of the virus and signaling the need for continued surveillance. To investigate the humoral responses to the virus in the context of epidemiologic settings in the epicenter of COVID-19 in China, we randomly selected individuals with or without suspected syndromes and convalescent COVID-19 patients from different consulting departments. Our results revealed a low positive rate of NA tests in the studied cohort. Seroprevalence for IgM and IgG in 355 individuals with suspected syndromes was significantly higher than those of 1295 asymptomatic participants (IgM and IgG seropositivities were 3.38% vs. 1.70% and 7.89% vs. 5.17%, respectively), indicating that viral infection in some cases has mild or even no clinical manifestations. IgM is considered a parameter of the early phase of infection. IgM against SARS-CoV can be detected as early as in the 1st week [16]. In our study, seroprevalence for IgM in ordinary patients for COVID-19 screening was more sensitive than the result of NA tests, which were almost all negative in our participants. The reason may be due to short phases of the virus shedding or insufficient sample quality which would decrease the chances of detecting nucleic acids [17]. IgM detection assays are incredibly valuable because they help to find patients in the acute phase and to elucidate the range of subclinically-infected individuals. In our study, the positive rate of IgM continues to decrease over time, consistent with a gradual decline in newly diagnosed cases. According to the seroprevalence for IgM and low positivity rate from NA testing, the SARS-CoV-2 outbreak is subsiding, and the possibility of reintroduction of the virus from an unidentified natural reservoir is low.

SARS-CoV-2 is transmitted by direct contact, droplets, feces, aerosols, or contaminated environmental surfaces [5]. Therefore, the higher risk faced by residents in the epicenter may be related in part to the temporal shedding pattern of the virus from COVID-19 patients. Seroprevalence for IgG remains in the range of less than 10% in Wuhan residents, which is much lower than we expected. The finding of such low seroprevalence may be attributed to strictly precautionary measures, including home quarantine, temperature measurement, wearing a surgical mask before entering public places, and washing hands frequently. The highest seroprevalence for IgG was observed in COVID-19 convalescents. However, the positive rate of IgG was only 77.27% in patients. Eleven patients were IgG-negative but IgM-positive, suggesting they were newly infected patients. Our data is consistent with the previous reports, in which the presence of antibodies was < 40% among patients within 1-week since onset and rapidly increased since day-15 after onset [18].

In sixteen medical staff who had suspected syndromes, the serological tests for IgM were entirely negative, the positive rate of IgG was 25%, and the positive rate of NA was 6.25%, indicating that latent infection had existed among the medical staff. Seroprevalence for IgG among the medical staff was significantly higher than those in Wuhan residents. Before the outbreak was recognized, COVID-19 was incredibly difficult to recognize due to the nonspecific nature of clinical manifestations, which may be the leading cause of high seropositivity amongmedical staff. After the outbreak was announced, all doctors entering isolation areas were required to follow an SOP regarding attire and to equip themselves with a double layer of personal protective equipment (PPE), including an N95 mask, covered with a full-face mask, goggles, a long-sleeve gown, a paper hat, and shoe covers. Our results showed that personal protection is effective against viral infection, even under high-risk viral exposure conditions.

NAbs are vital components in the protective immune response to viral infections [11, 19]. When evaluating the impact of comorbidity on immune responses, higher seroprevalence was observed in patients with tumors, cardiovascular and cerebrovascular diseases, hypertension, diabetes, nervous system disease, and digestive system diseases, but there was no significant difference among the various groups. Seropositivity was higher in advanced age participants, and low in the younger populations. Females had a higher seroprevalence of IgG than males, which may be due to low adaptive immune response in men relative to women [20]. A significant finding of our study is that none of the AIDS patients were infected. Both IgM and IgG were negative. Since there were only 13 patients, we cannot rule out a sampling error. However, three possible reasons are that [21]: (1) as a group of individuals with low immunity, AIDS patients pay close attention to protection against infection in their daily life. (2) AIDS patients have poor immune systems, so the negative response to SARS-CoV-2 may due to their immunocompromised immune system and weak ability to produce antibodies and (3) The anti-viral durgs, such as Lopinavir and Ritonavir, protect AIDS patients from infection. Regarding COVID-19, information on immunity and pathogenesis is insufficient to provide a comprehensive basis for a specific drug or vaccine design. The observational data of NAbs may provide leads in controlling the possible reemergence of the disease.

Based on the transmission risk of known or unknown sources, infectious sources could not be ascertained. Patients without suspected syndromes were significantly more likely to be seronegative than those with the suspected syndrome. Moreover, participants from the fever clinic had a significantly higher positive rate of IgM and IgG than other departments, demonstrating that the establishment of a fever clinic for screening suspected cases can improve the detection rate and achieve the goal of first isolation. The finding in asymptomatic seropositive persons indicates that the test will be useful in more extensive retrospective surveillance studies, which are needed to define the epidemiology and spectrum of disease fully [22].

## Conclusion

In the later period of the epidemic, the potential reintroduction of the virus from an unidentified natural reservoir remains a concern. Serosurveillance is particularly valuable to trace hotspots of persons carrying antibodies to SARS-CoV-2 and to track the origins of the disease. The low rate of SARS-CoV-2 antibodies in Wuhan residents indicates that the chain of human transmission can be successfully interrupted by public health intervention.

## Data Availability

The datasets used and/or analysed during the current study are available from the corresponding author on reasonable request.

## Abbreviations

COVID-19: Coronavirus disease 2019
SARS-CoV-2: Severe acute respiratory syndrome coronavirus-2
R0: Basic reproduction number
Nabs: Neutralizing antibodies
IgM: Immunoglobulin M
IgG: Immunoglobulin G
NA: Nucleic acid
RT-PCR: Reverse transcription-polymerase chain reaction
CCD: Cardiovascular and cerebrovascular diseases, hypertension, and diabetes
COPD: Chronic obstructive pulmonary diseases
SOP: Standard operation procedure
PPE: Personal protective equipment
AIDS: Acquired immune deficiency syndrome
